# Recent Status of Phase I Clinical Trials for Brain Tumors: A Regulatory Science Study of Exploratory Efficacy Endpoints

**DOI:** 10.1007/s43441-024-00644-3

**Published:** 2024-03-26

**Authors:** Shinya Watanabe, Takahiro Nonaka, Makoto Maeda, Masanobu Yamada, Narushi Sugii, Koichi Hashimoto, Shingo Takano, Tomoyoshi Koyanagi, Yoshihiro Arakawa, Eiichi Ishikawa

**Affiliations:** 1 Department of Neurosurgery, Mito Kyodo General Hospital, Tsukuba University Hospital Mito Area Medical Education Center, 3-2-7 Miyamachi, Mito, 310-0015 Ibaraki Japan; 2 Department of Neurosurgery, Institute of Medicine, University of Tsukuba, Tsukuba, Japan; 3 Department of Health and Medical Innovation, Graduate School of Medicine, Osaka Metropolitan University School, Osaka, Japan; 4 Department of, Pharmacy, National Cancer Center Hospital, Tokyo, Japan; 5 Department of Neurosurgery, University of Tsukuba Hospital, Tsukuba, Japan; 6 Tsukuba Clinical Research and Development Organization, University of Tsukuba, Tsukuba, Japan

**Keywords:** Brain tumors, Efficacy endpoint, Phase I clinical trial, Regulatory science, Meningioma, Glioblastoma

## Abstract

**Background:**

Appropriate exploratory efficacy data from Phase I trials are vital for subsequent phases. Owing to the uniqueness of brain tumors (BTs), use of different strategies to evaluate efficacy is warranted. We studied exploratory efficacy evaluation in Phase I trials involving BTs.

**Methods:**

Using Clarivate’s Cortellis^™^, 42 Phase I trials of BT interventions conducted from 2020 to 2022 were analyzed for efficacy endpoints, which were set as primary endpoints (PEs) or secondary endpoints (SEs). Additionally, these metrics were compared in two subgroups: trials including only BTs (Group-A) and those including BTs among mixed solid tumors (Group-B).

**Results:**

Selected studies included a median of 1.5 PEs (range, 1–6) and 5 SEs (range, 0–19). Efficacy endpoints were included as PEs and SEs in 2 (5%) and 31 (78%) trials, respectively. Among the latter 31 trials that included 94 efficacy endpoints, 24, 22, 20, 9, and 8 reflected overall response rate (ORR), progression-free survival (PFS), overall survival (OS), duration of response (DOR), and disease control rate (DCR), respectively. ORR for BT was determined using various methods; however, the Response Evaluation Criteria in Solid Tumors (RECIST) was used less frequently in Group-A than in Group-B (*p* = 0.0039).

**Conclusions:**

Recent Phase I trials included efficacy endpoints as SEs, with ORR, PFS, or OS included in ~ 50% trials and DOR or DCR in ~ 25%. No established criteria exist for imaging evaluation of BTs. Phase I trials involving mixed solid tumor cohorts revealed challenges in designing methods to assess the exploratory efficacy of BTs.

## Introduction

Phase III clinical trials of anticancer drugs usually include overall survival (OS) and (or) progression-free survival (PFS) as primary endpoints (PEs) [1]. However, in earlier stages of clinical development such as Phase I and II, the choice of efficacy endpoint and inclusion as a PE or secondary endpoint (SE) varies substantially [1, 2]. For instance, the inclusion of exploratory efficacy evaluation in Phase I trials and surrogate efficacy evaluation in Phase II may depend on whether the study involves solid tumors other than brain tumors (BTs) or only BTs. For BTs, such as glioblastoma and malignant meningioma, the development of treatment methods has proven particularly difficult [3–5]. However, there are currently several challenges to setting better efficacy endpoints in Phase I and Phase II trials.

In Phase II trials for solid tumors, other than BTs, the Response Evaluation Criteria in Solid Tumors (RECIST) [6] is well established as an imaging-based efficacy endpoint and has been used in > 90% of Phase II trials to evaluate surrogate efficacy [7]; however, there is no consensus on the appropriateness of the RECIST for evaluating the surrogate response rate of BTs, such as glioblastoma or meningioma [7, 8]. Therefore, various efficacy endpoints have been selected in Phase II trials for BTs, such as OS, PFS, other time-to-endpoint indices, and overall response rate (ORR). The situation regarding the ORR is chaotic, with various indicators, such as Response Assessment in Neuro-Oncology (RANO) criteria [9], immunotherapy RANO criteria [10], as well as RECIST mentioned earlier, being used. Ultimately, international regulatory harmonization has not yet progressed on a surrogate efficacy endpoint.

In our previous study, we indicated that the following issues needed to be addressed for imaging evaluation in BTs; these include evaluation using RECIST, as this is a one-dimensional imaging evaluation method while lesions after standard treatment (surgical resection) are irregular due to anatomic limitations [7]. Moreover, image modifiers such as pseudoprogression [8] and radiation necrosis [11] after standard multidisciplinary treatment make it difficult to evaluate efficacy based on imaging alone. Additional hurdles include diverse tumor classifications [12], the limited number of responders [13] during early trial phases, and small sample sizes due to the general rarity of these conditions [14, 15]. Given the unique evaluation methods and criteria used for BTs, regulatory science research on trials including BTs is needed to reach a consensus on efficacy endpoints.

In terms of Phase I trials, to increase the probability of success in Phase III trials or of conditional early approval based on Phase II trial results alone, it is vital that recent Phase I trials obtain appropriate exploratory efficacy data, in addition to safety and tolerability results, for subsequent phases [16]. Unlike Phase II trials, Phase I trials for BTs are generally included in trials together with other solid tumors during the development of anticancer treatment [17–19]. However, BTs are unique in many ways, and biological factors, such as the blood–brain barrier and the unique tumor and immune microenvironment, are significant challenges in the development of novel therapies. Innovative clinical trial designs with biomarker-enrichment strategies are required to improve the outcome of patients with glioblastoma [20]. Therefore, necessitating different exploratory efficacy evaluation strategies would be needed, and there are potentially more complex situations in Phase I trials than that in Phase II.

Therefore, we investigated the status of exploratory efficacy evaluation in Phase I clinical trials for BTs and addressed such issues as frequency of inclusion among PEs or SEs and the specific parameters used, such as ORR, PFS, OS, duration of response (DOR), and disease control rate (DCR). We also examined if efficacy evaluation differs between trials exclusively on BTs and trials of mixed solid tumor cohorts including BTs.

## Materials and Methods

To survey exploratory evaluation methods used in recent Phase I clinical trials of BTs, Clarivate’s Cortellis™ Clinical Trial Intelligence was used. Regarding the inclusion criteria, a database search using the terms “brain tumor” and “Phase I trials” during the period “April 1, 2020 to March 31, 2023” was performed. This search identified 58 Phase I trials that involved BTs, with several trials excluded based on specific criteria. Subsequently, the characteristics of the selected trials, including types of treatment, region, organization, and other relevant factors, were summarized.

The primary purpose of the study was to examine PE and SE settings in Phase I clinical trials, focusing on exploratory efficacy assessment. These endpoints were evaluated and analyzed in the overall population and in two subgroups: trials that included only BTs (Group-A) and those that included BTs among mixed solid tumors (Group-B).

For baseline variables, summary statistics were constructed using frequencies and proportions for categorical data and medians, means, and standard deviations for continuous variables. All analyses were conducted using JMP version 10.0.0 (SAS). The Wilcoxon test was used to analyze continuous variable data, and the Fisher’s exact test was used to analyze categorical data. A p-value of < 0.05 was considered significant for all statistical tests.

## Results

**Fig. 1:**
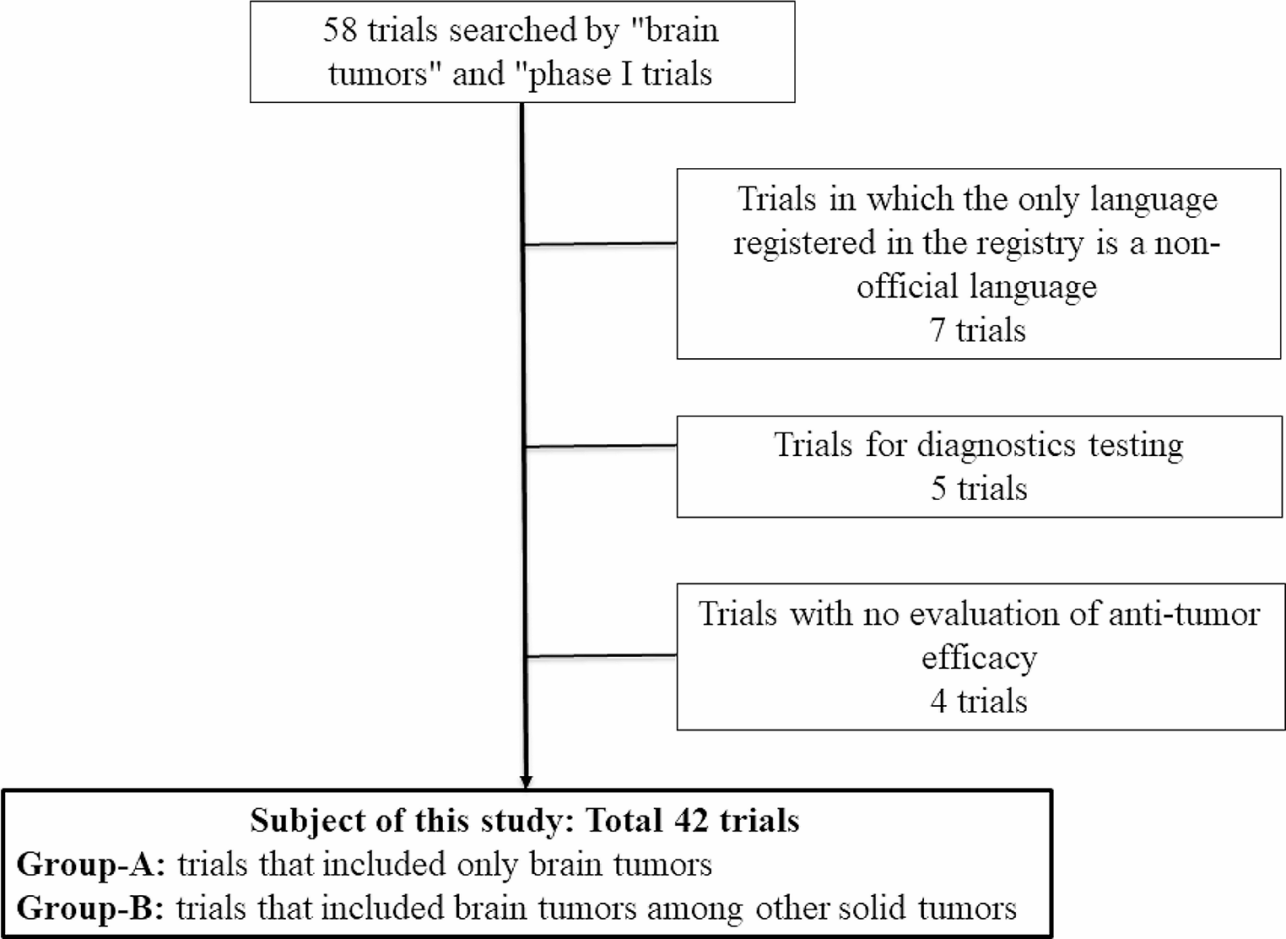
Flowchart of inclusion and exclusion criteria for Phase I clinical trials including brain tumor

Of the 58 trials identified, 16 were excluded (7 because the enrollment registry language was not an official language for English, 5 diagnostics trials, and 4 trials because there was no evaluation of anti-tumor efficacy) (Fig. 1). Background information on the remaining 42 studies is summarized in Table 1. The largest proportion of these trials tested pharmaceutical products, followed by cell therapies, combination therapies, radiotherapies, a medical device, and a vaccine trial. Most were conducted in the United States. Multiple studies were also conducted in China and Iran, whereas the rest were from nine individual countries. The majority were conducted in academia, either exclusively or in collaboration with industry or the government. The number of study sites (median, min–max.) was 1 (1–21) and the number of subjects to be included in each study (median, min–max.) was 27 (1–292). Most BTs included in this study were malignant, such as glioblastoma and metastatic BTs.

**Table 1:** Background information on the Phase I clinical trials included in this study (*N* = 42)

Item	Category	Value
Treatment	Pharmaceuticals	25 trials (60%)
	Cell therapies	6 trials (14%)
	Multiple combinations	6 trials (14%)
	Radiotherapies	3 trials (7%)
	Medical devices and vaccines	1 trial each (2% each)
Region^*^	USA	23 trials (56%)
	China	7 trials (17%)
	Iran	2 trials (5%)
	Others	9 trials (2% each)
Organization(s)	Academia	16 trials (38%)
	Company	11 trials (26%)
	Academia + government	6 trials (14%)
	Academia + industry	4 trials (10%)
	Government	3 trials (7%)
	Academia + government + industry	2 trials (5%)

*No information for one study (probably USA)

**Fig. 2:**
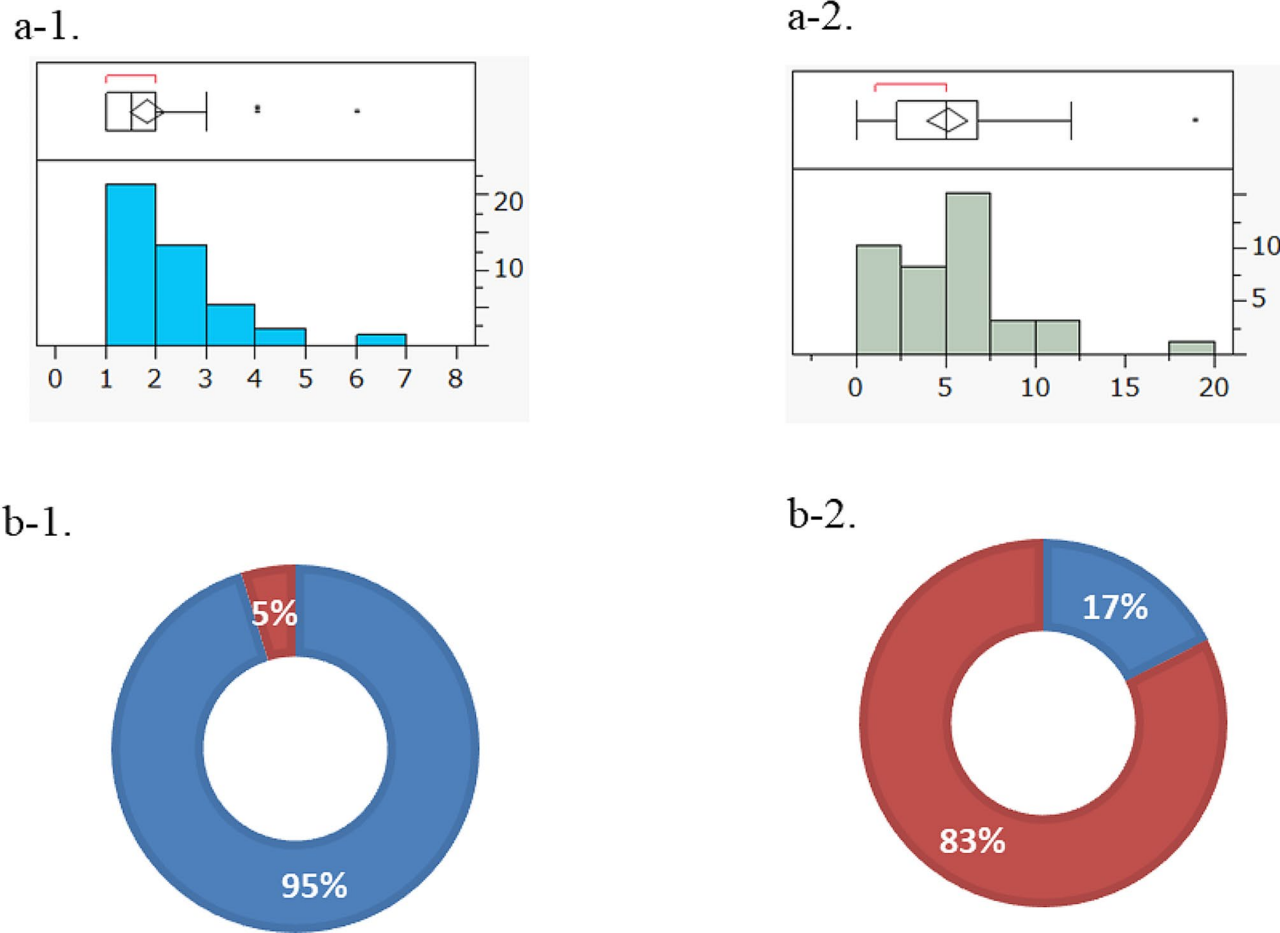
Exploratory efficacy endpoints in the 42 Phase I clinical trials selected **a-1.** Numbers of primary endpoints (PEs) set Median 1.5 (range, 1 to 6) **a-2.** Numbers of secondary endpoints (SEs) set Median 5 (range, 0 to 19) *Two of the 42 trials set no SEs, so the population was 40 trials **b-1.** Efficacy endpoint types set as PEs. Blue: no efficacy endpoint, red: efficacy endpoint established Two trials (5%) included an efficacy endpoint as a PE, in both cases overall response rate (ORR). **b-2.** Efficacy endpoint types set as SEs. Blue: no efficacy endpoint, red: efficacy endpoint established Thirty-one trials included efficacy endpoints as SEs (78%) *Two of the 42 trials set no SE, so the population was 40 trials

For all included 42 trials, the median number of PEs was 1.5 (range, 1–6) and the median number of SEs was 5 (range, 0–19) (Fig. 2a-1, -2). Only 2 trials (5%) included an efficacy endpoint as a PE (ORR in both trials). In contrast, 31 trials (78%) included an efficacy endpoint as an SE (Fig. 2b-1, -2). Of the 31 trials, 24 included ORR as the most frequent endpoint. Of these 24 trials, 15 defined ORR according to the RECIST one-dimensional imaging endpoint and 5 according to the RANO two-dimensional imaging endpoint. Additionally, there were a total 22 PFS, 20 OS, 9 DOR, 8 DCR, 3 quality of life, and 8 others with efficacy endpoints as SEs (Table 2).

**Table 2:** Breakdown of the total number of established valid efficacy endpoints in the trials with efficacy endpoints

Item	Endpoint category	Number
Primary endpoints (*N* = 2)	ORR	2
Secondary endpoints (*N* = 94)	ORR^*^	24
	PFS	22
	OS	20
	DOR	9
	DCR	8
	QOL	3
	Others	8

ORR, overall response rate; PFS, progression-free survival; OS, overall survival; DOR, duration of response; DCR, disease control rate; QOL, quality of life

^*^Response Evaluation Criteria in Solid Tumors (RECIST) 15, Response Assessment in Neuro-Oncology (RANO) 5, others 4

**Fig. 3:**
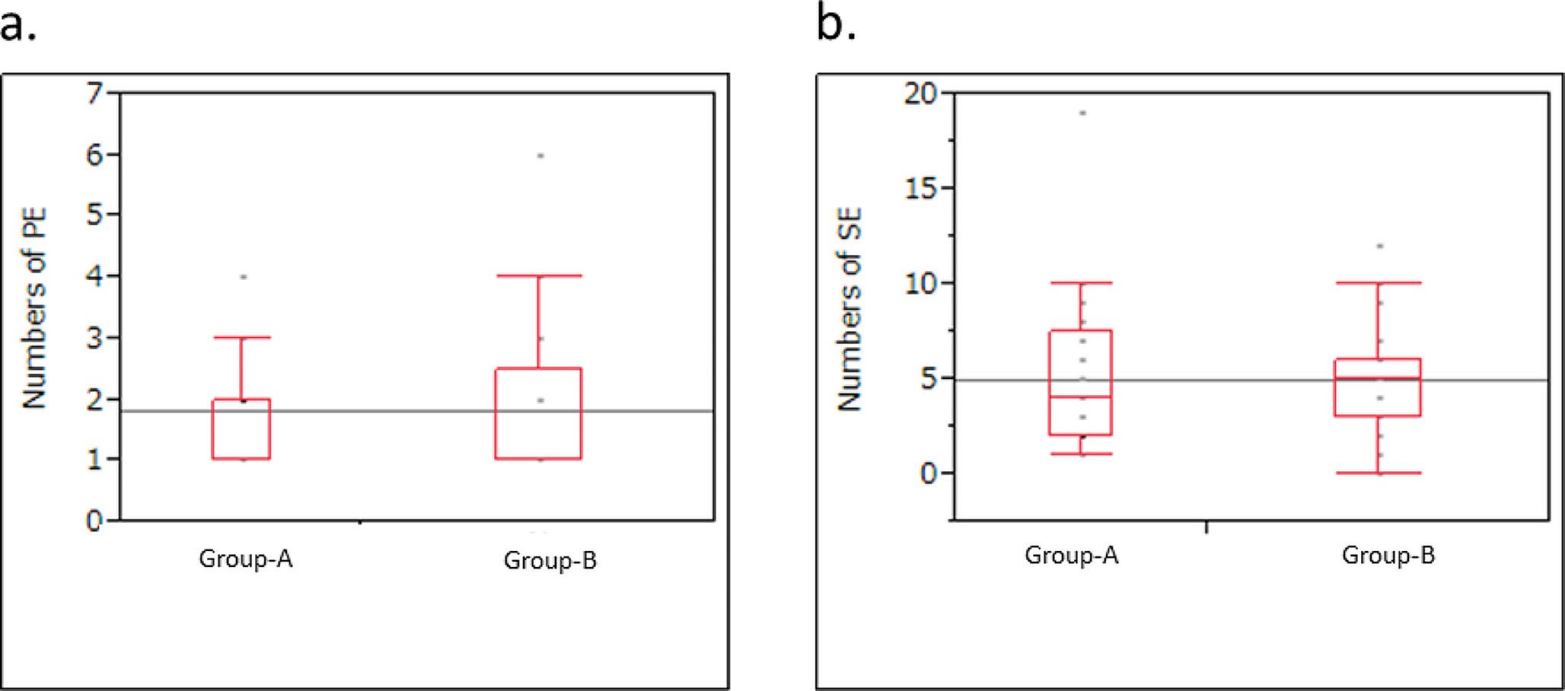
Comparison of primary and secondary endpoints between two groups: trials examining brain tumors (BTs) only (Group-A, 17 studies) vs. trials examining multiple solid tumors including BTs (Group-B, 25 studies)**a.** The median number of primary endpoints per study did not differ between groups **b.** The median number of secondary endpoints per study did not differ between groups

**Fig. 4:**
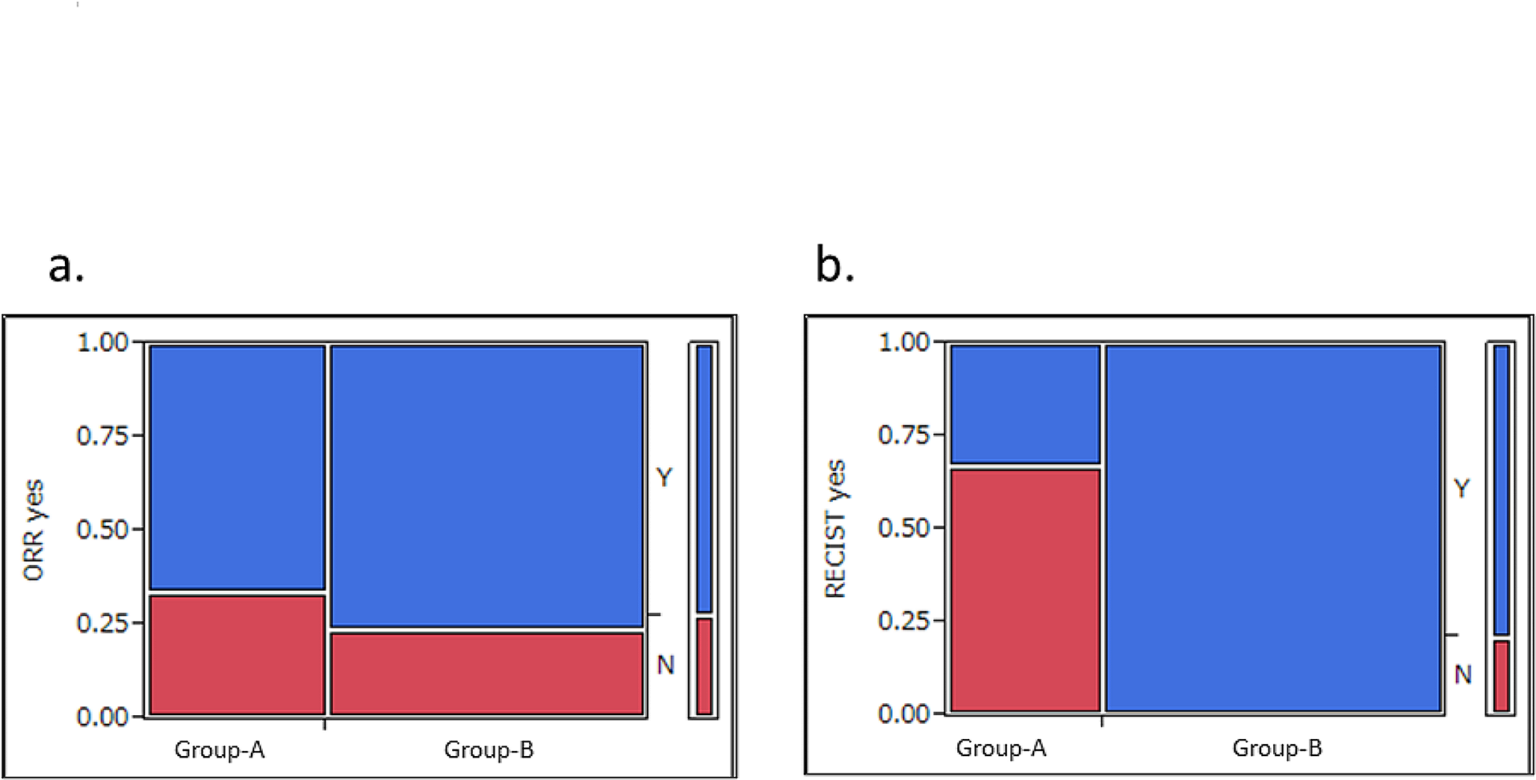
Analyses of 33 trials that set efficacy endpoints as either PE or SE: trials examining brain tumor only (Group-A, 12 studies) vs. trials examining multiple solid tumors including brain tumor (Group-B, 21 studies)**a.** Use of overall response rate (ORR) as an endpoint by trials**b.** Use of RECIST as an ORR endpoint by trials

For two-subgroup analysis, seventeen trials studied BTs only (Group-A) while 25 enrolled solid tumor patients including some BTs (Group-B). The median number of PEs did not differ between Group-A and Group-B (2 [range, 1–4] vs. 1 [range, 1–6]; *p* = 0.72) (Fig. 3a). Similarly, the median number of SEs was not significantly different (4 [1–19] vs. 5 [0–12]; *p* = 1.00) (Fig. 3b). Of the 33 trials that set efficacy endpoints as either PE or SE, most in both Group-A and -B used ORR without a significant group difference (8/12, 67% vs. 16/21, 76%; *p* = 0.69) (Fig. 4a). However, among various ORRs, RECIST was used significantly more often in Group-B compared to Group-A (*p* = 0.0039) (Fig. 4b).

## Discussion

Anticancer drug development has changed dramatically in recent years, largely due to the success of molecularly targeted drugs such immune checkpoint inhibitors and others targeting important driver mutations [1]. It is therefore essential that clinical trials are designed to facilitate more timely regulatory approval while maintaining patient safety. Critical to this goal is the inclusion of multiple exploratory efficacy endpoints in early phase, i.e., Phase I trials. According to the present survey, the role of academia appears to be increasing in early phase, in accordance with recent global trends [21]. Collectively, these research trends underscore the importance of documenting efficacy outcomes as early as possible during the development process. Strategies to accelerate clinical development for solid tumors other than BTs had been advanced, while the special characteristics of BTs have hindered parallel progress. Nonetheless, there have been no detailed analyses of exploratory efficacy assessment methods used in Phase I trials for BTs. Thus, we conducted a detailed analysis for creating consensus of appropriate exploratory efficacy endpoint setting in Phase I trials for BTs. In this study, we showed that Phase I trials had exploratory efficacy in SE in as many as 78% of the trials, which clearly indicated the importance of early efficacy evaluation. This study also revealed that trials that incorporated BTs along with mixed solid tumors significantly often employed RECIST, a one-dimensional imaging measure that is not suitable for assessing BTs.

### Significance of Exploratory Efficacy Endpoints in Phase I Clinical Trials Including BTs

Despite this greater focus on earlier efficacy evaluation, we previously found that there is still no consensus on appropriate surrogate efficacy endpoints in Phase II clinical trials on glioblastoma treatment [7]. Although surrogate efficacy endpoints related to time-to-event were used, such as OS and PFS, some trial designs made it difficult to interpret these clinical trial outcomes. In another one of our studies [8], we also found that various time-to-event efficacy endpoints were set for Phase II clinical trials for meningioma. In most cases, these time-to-event endpoints were compared to clinical data from previous studies (due for instance to the absence of an internal control group). Therefore, in cases of setting time-to-event endpoints, it might be essential to accumulate detailed prognostic data for standardization of time-to-event efficacy endpoints.

Alternatively, the main purpose of Phase I trials is to evaluate the safety and tolerability to prepare for Phase II, and indeed, most PEs were related to safety and tolerability, whereas our study showed only two trials included an exploratory efficacy endpoint as PEs. However, in our study, most (78%) did include at least one efficacy endpoint as SEs, suggesting that the designers of Phase I trials, including BT treatment, were attempted to facilitate faster phase progression or earlier approval. In addition, it is noteworthy that DOR or DCR was included as an efficacy endpoint in some Phase I trials because DOR or DCR was rarely set in Phase II trials, as reported in our previous studies [7, 8]. Our result emphasized the importance of comprehensive efficacy endpoint evaluation in many recent Phase I trials.

### Differences in Exploratory Efficacy Evaluation Design between Phase I Trials Involving Only BTs and Trials Including Mixed Solid Tumors

Phase I trials that included only BTs and those that included BTs among mixed solid tumors (termed Group-A and Group-B) did not differ in number of PEs or SEs, suggesting complexity of the target disease does not influence endpoint setting. Further, among the trials with efficacy endpoints as PEs or SEs, ORR was the most frequently used in both groups. However, RECIST for ORR was used more extensively in studies of mixed solid tumors, suggesting continued difficulty evaluating the response of BTs using one-dimensional imaging evaluation and the need for specific indices tailored to individual BT types. The RANO group has proposed methods to evaluate response according to tumor and treatment type, including glioblastoma [9] in 2010, evaluation after immunotherapy [10] in 2015, brain metastases [22] in 2015, and meningiomas [23] in 2019. Therefore, BTs may ideally require individualized evaluation based on type. However, most Phase I trials of mixed solid tumor cohorts were designed using evaluation methods, such as RECIST, originally developed for solid tumors outside the brain [6].

### How Should the Exploratory Efficacy of BTs be Assessed in Phase I Trials?

Some Phase I trials of treatments for BTs used exploratory efficacy endpoints adapted to those used for solid tumors other than the brain, and much less frequent use of RECIST was shown in the present study. Therefore, it might be therefore necessary to establish response evaluation criteria or to use time-to-event endpoints for which historical data are sufficiently stable types of BTs. Regarding imaging evaluation of BTs when they are incorporated along with other solid tumors, to account for the specificity of BTs, it might be acceptable to use a unique imaging metric rather than a one-dimensional metric to account for their specificity. Although BTs are included in clinical trials involving other solid tumors, the protocol should specify in advance that researchers will evaluate BTs using metrics specific to BTs, rather than the metrics used for other solid tumors.

### Strengths and Limitations

This is the first study to evaluate exploratory efficacy endpoints in Phase I clinical trials for BTs, contributing to the establishment of a consensus on appropriate endpoints for BTs. However, this study had some limitations. First, the primary objective of this study was ideally to determine the best exploratory efficacy endpoints. However, the best endpoints may vary with the aim of each clinical trial. Therefore, additional research is warranted to assess the endpoints that are most appropriate for specific long-term aims. The nature of the study and lack of detailed information on the type of BTs was available are also a limitation of the study. Second, a single database was used because we excluded trials using non-English languages. We could not compare our results with those of other papers, because there is little supporting literature. Therefore, we will aim to continuously accumulate evidence internally. Third, the duration and costs of these trials were not determined. In future studies, we will analyze long-term global information using contracted database information to compare duration, cost, rate of progression to the next phase, and Phase III success rates according to the early efficacy endpoints.

In addition, New WHO brain tumor guidelines [12] have recently been issued, so evidence based on such classification should be accumulated for these analyses for the future. It may be necessary for future studies to consider methods for individualized efficacy evaluation based on new classifications, such as by gene mutation.

## Conclusion

Most recent Phase I trials of anticancer treatments for BTs have reported exploratory efficacy evaluations such as ORR, PFS, and OS among the secondary endpoints, while a minority have reported DOR or DCR. However, the use of RECIST for efficacy evaluation remains low in Phase I trials of BTs treatments compared to trials assessing treatments for multiple solid tumors including BTs. Thus, multidimensional criteria are still required for expanded use of efficacy evaluation in early-phase trials of brain tumor treatments. It is essential to define improved tumor type-specific imaging criteria for ORR evaluation of brain tumor to facilitate treatment development.
